# Single-fiber EMG: A review

**DOI:** 10.4103/0972-2327.78058

**Published:** 2011

**Authors:** V. Arul Selvan

**Affiliations:** Walton Center for Neurology and Neurosurgery, Lower Lane, Fazakerley, L9 7LJ, Liverpool, UK

## Introduction

Single fiber electromyography (SFEMG) was established by Stalberg and Eskedt in the 1960s, and is of proven value in the diagnosis of neuromuscular disorders, especially myasthenia gravis.[1] It has proved to be the most sensitive technique in detecting a neuromuscular transmission defect in comparison with the tensilon test, repetitive stimulation, and acetyl choline receptor antibody estimation.[2,3]

**Figure 1:**
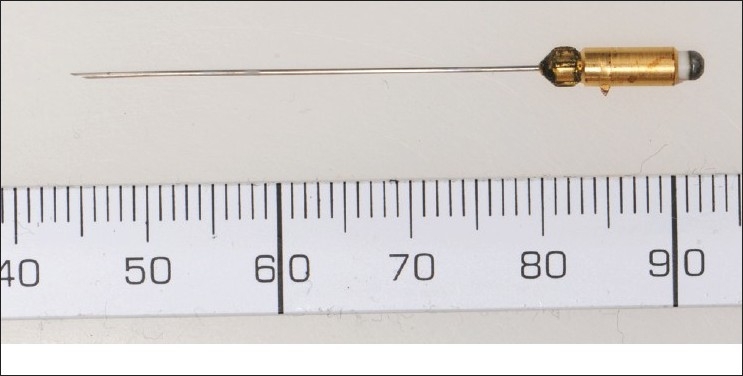
Single fibre needle electrode

**Figure 2:**
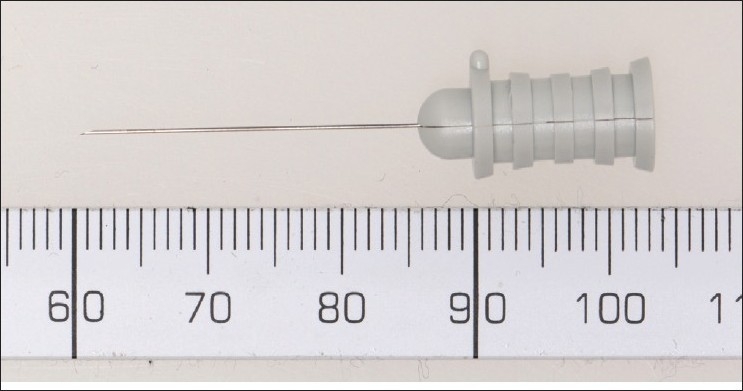
Concentric facial needle electrode

Single fiber electromyography typically requires the use of a specially contracted single fiber EMG needle electrode[Figure 1] or facial concentric needle electrode [Figure 2] with a small recording surface (25 micrometers), which is exposed at a port on the side of the electrode, 3 mm from the tip.

**Figure 3:**
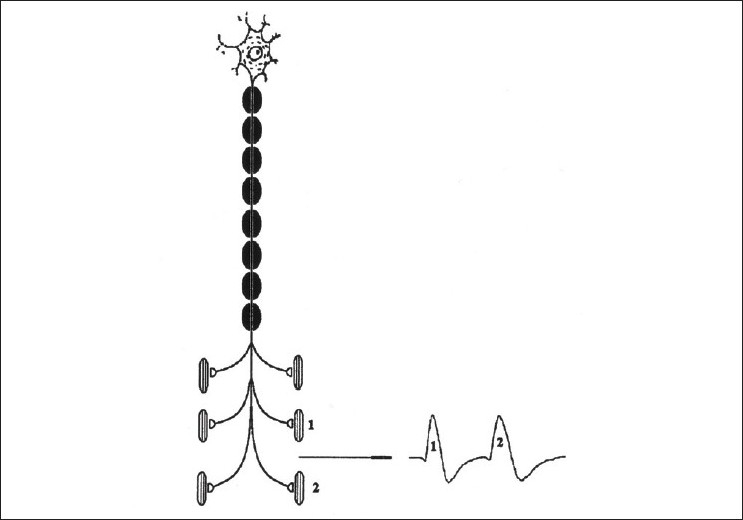
A single fiber EMG needle placed between two individual muscle fibers can record the variation of the interpotential intervals of two individual muscle fibers of the same motor unit.(With permission from EMG and Neuromuscular Disorders, Preston and Shapiro; Butterworth – Heinemann)

The validity of the technique has been proven by examining a large number of myasthenia patients with a sensitivity of up to 99% in detecting a neuromuscular transmission defect. in generalized myasthenia gravis has been reported.[2,4] When a motor axon is depolarized the action potentials travel distally and excite the muscle fiber more or less at the same time. The variation in the time interval between the two action potentials of the same motor unit is called as “jitter”. SFEMG measures the variation of this inter potential interval (jitter)[Figure 3].

### Technical considerations

Most of the modern nerve conduction and EMG machines have a software to perform and analyze the SFEMG examination. There are two methods to perform this. One is stimulated and the other is under a volitional effort. However, most physicians, including the author, prefer to perform SFEMG under the volitional effort. The goal of SFEMG is to study the adjacent action potentials from the same motor unit, known as ‘pairs’. This is achieved by using a specially constructed single fiber needle electrode or facial concentric needle electrode. This identifies and selectively records the action potentials from individual muscle fibers. The selectivity of the recording is further strengthened by adjusting the filter settings. (Low frequencies filter 500 HZ – high frequencies filter 10 KHZ). This filter setting selectively abolishes low frequency components from distant muscle fibers. Action potentials should be greater than 200 mictovolts in amplitude and the rise time should be less than 300 microseconds. Around 20 potential pairs are collected from the same muscle by three-to-four insertions. The subject is asked to maintain a steady contraction if volitional SFEMG is undertaken, until 100 consecutive discharges are recorded from each pair.

Stimulation SFEMG is particularly useful in children, uncooperative, comatose patients, and those who have tremors. A branch of the motor nerve is stimulated by using a mono polar needle electrode and recording is made by SFEMG or a concentric needle electrode. Stimulation is delivered at 2 - 10 HZ and the stimulus intensity is adjusted accordingly.

### Jitter Analysis:

**Figure 4:**
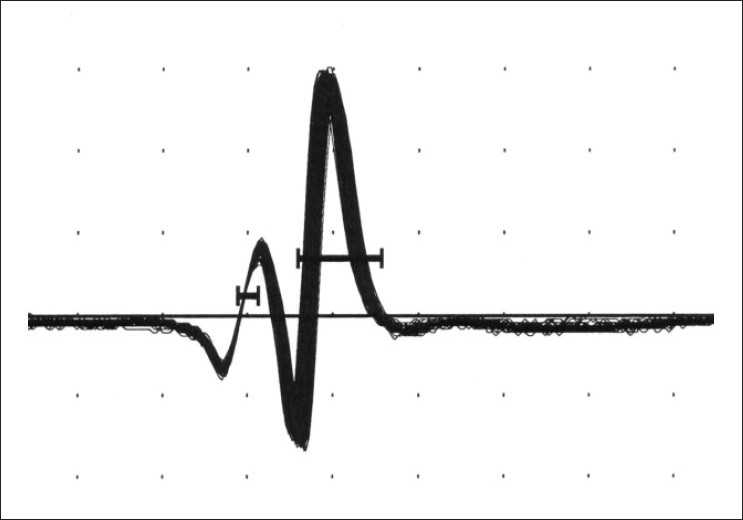
Normal Jitter values (100 traces superimposed)

**Figure 5:**
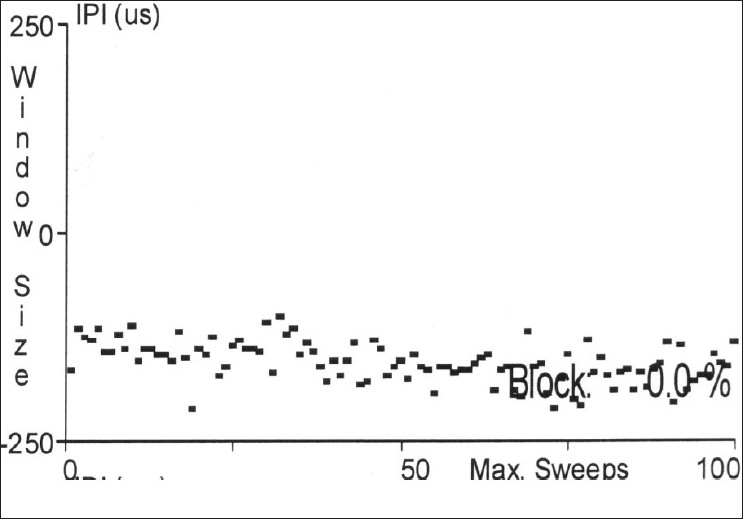
Normal Jitter values in a sequential plot

**Figure 6:**
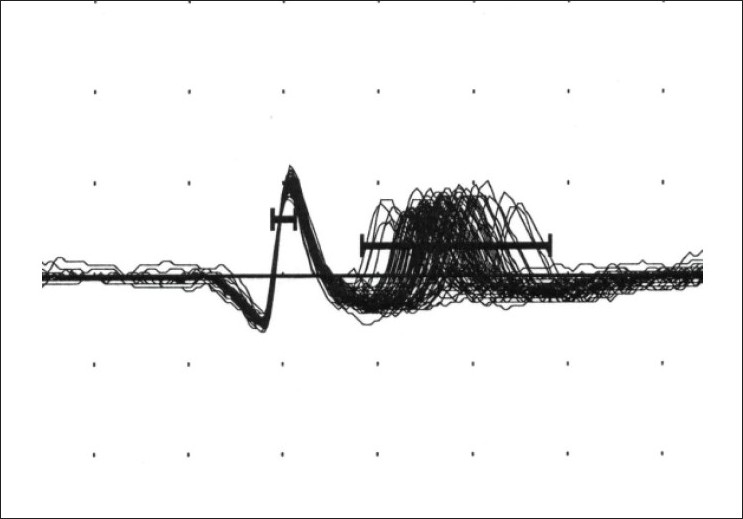
An abnormal Jitter value (100 traces superimposed)

**Figure 7:**
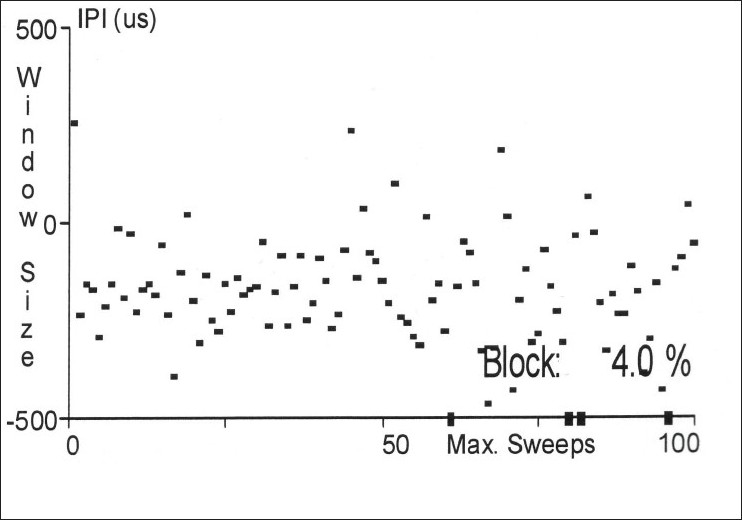
Abnormal jitter values in a sequential plot

Jitter is the measurement of variation of the inter-potential interval. This is calculated between the triggered potential and the time-locked, second single muscle fiber action potential [Figures 4–7]. This is expressed as a mean consecutive difference (MCD). Most modern EMG machines have a program that automatically performs the MCD calculation. Mean MCD is calculated using the following formula:

$$\mathrm{MCD}\;=\;\left(IP1\;-\;IP2\right)\;+\;\left(IP2\;-\;IP3\right)\;+\;\left(IPn\;-\;IPn\right)/n-1$$

**Figure 8:**
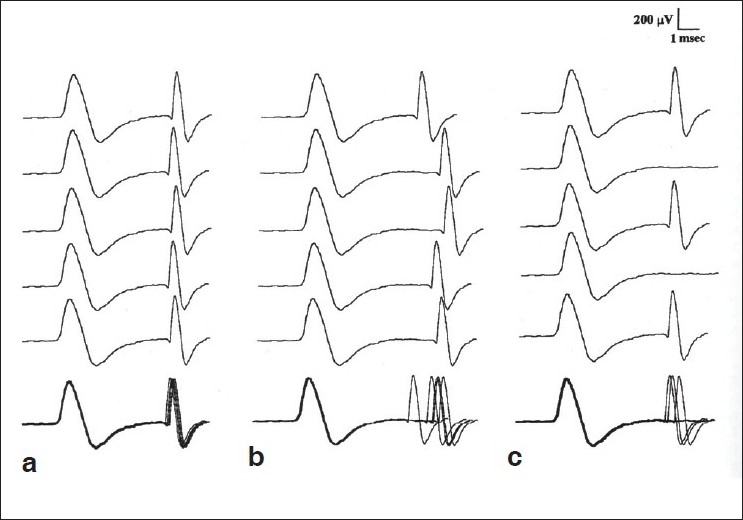
Single fiber EMG recordings: (a) Normal; (b) Increased; Jitter (c) Blocking both increased jitters, and blocking is seen in the neuromuscular disorders. (With permission from EMG and Neuromuscular Disorders, Preston and Shapiro; Butterworth - Heinemann)

When neuromuscular transmission is sufficiently impaired, nerve impulses fail to elicit an action potential and this is called ‘blocking’ [Figure 8]. This usually happens when the jitter value is markedly prolonged, usually when MCD is more than 100 microseconds.[5]

### Normal values

**Figure 9:**
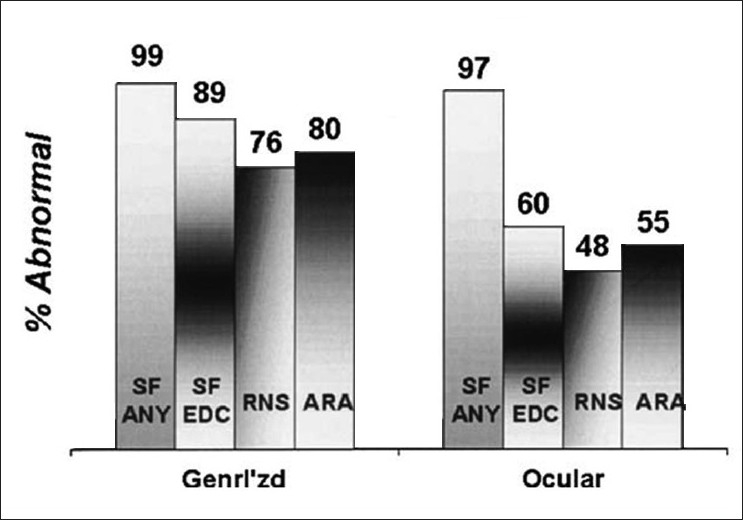
Sensitivity of the diagnostic procedures in 550 untreated myasthenia gravis patients. The percentage of patients with abnormal results was noted in each technique. SF ANY - single fiber studies were abnormal in at least one muscle; SF EDC - SFEMG studies were abnormal in the extensor digitorum communis muscle; RNS - repetitive nerve stimulation demonstrated a decrement in a hand or shoulder muscle; ARA - AChR antibody was abnormal (with permission from D.B.Sanders, J.M.Massey, J.F. Howard – unpublished)

The normal jitter values have been determined for many muscles in a multicenter collaborative study[6,7][Figure 9].

The study is considered abnormal if one of the following criteria is met,

- Mean jitter value exceeds the upper limit of the normal value
- More than 10% of the pairs have increased jitter (two out of twenty pairs)

Normal values apply only if the inter-spike interval is up to four microseconds. Errors may be encountered if this is higher and may produce a false jitter.

## Discussion

In patients with myasthenia gravis, jitters are greater in the weak muscles, but they are also increased in muscles with normal strength. Jitter is abnormal in the orbicular oculi > 95%, followed by frontalis and extensor digitorum communis in 85% of the patients. Jitter is abnormal even when patients take anticholinesterase inhibitors.[8] It is our practice to not recommend stopping this, except when the study is normal and also when there is a strong clinical suspicion of myasthenia gravis. Extensor digitorum communis is usually tested first, unless symptoms or signs are limited to extraocular muscles when orbicicularis oculi or frontalis are tested.

Jitter is increased in myasthenia gravis, but it does not correlate well with disease severity.[8,9] However, in serial SFEMG studies, the mean jitter values increase by at least 10% in the tested muscles in two-thirds of the patients who become worse, and the converse is also true.[8,9] In a few cases, SFEMG was performed before and after remission of myasthenia. Although the mean jitter values had decreased, some pairs still showed abnormalities, indicating that SFEMG did not normalize completely.

A comparison was made between the diagnostic yield of repetitive stimulation, antibody titers, and SFEMG [Figure 10]. The SFEMG was highly sensitive (99%), followed by the acetyl choline receptor antibody, and the least sensitive was repetitive stimulation (76%), if the proximal muscles were tested.[1] Repetitive stimulation was technically difficult and mild decremental response was well-recognized in motor neurone disease and peripheral neruopathies.[10] In myasthenia gravis, the decremental response was less pronounced in the distal muscles than in the proximal muscles. Acetyl cholinereceptor antibody was detected in only 50% of ocular myasthenia and 85% of generalized myasthenia patients.[11,12] The remaining patients were treated as ‘seronegative’, but a proportion of such patients have antibodies to MUSK (muscle specific tyrosine kinase).

Single fiber electromyography is highly sensitive, but not specific to the diagnosis of myasthenia and myasthenic syndromes. It must be emphasised that increased jitter values are not pathogonomic for myasthenia, but indicate disturbed neuromuscular transmission. Increased jitter values are seen during the early stages of reinnervation, when motor unit remodeling occurs. Such changes can be seen in motor neurone disease, polyneuropathies, polymyositis, and Facioscapulohumeral dystrophy. However, it is also true that if SFEMG is normal in a weak muscle, it almost completely excludes the diagnosis of myasthenia.

## Conclusion

Single fiber electromyography is the most sensitive test to demonstrate an impaired neuromuscular transmission like myasthenia gravis.[13] However, it must be emphasized that it is not specific, as SFEMG can be abnormal in other myopathic and neuropathic disorders. The test is safe, but technically demanding for both the patient and the neurologist performing it. It needs considerable experience and technical expertise.[14]

**Table 1:** Reference values for Jitter measurement in healthy subjects during voluntary muscle activation (microseconds): 95% confidence limits for upper limit of mean, MCD / 95% confidence limits for MCD values of individual fibers[2]

Muscle	10yr	20yr	30yr	40yr	50yr	60yr	70yr	80yr	90yr
Frontalis	33.6/49.7	33.9/50.1	34.4/51.3	35.5/53.5	37.3/57.5	40.0/63.9	43.8/74.1		
Obicularis oculi	39.8/54.6	39.8/54.7	40.0/54.7	40.4/54.8	40.9/55.0	41.8/55.3	43.0/55.8		
Obicularis oris	34.7/52.5	34.7/52.7	34.9/53.2	35.3/54.1	36.0/55.7	37.0/58.2	38.3/61.8	40.2/67.0	42.5/74.2
Tongue	32.8/48.6	33.0/49.0	33.6/50.2	34.8/52.5	36.8/56.3	39.8/62.0	44.0/70.0		
Stern Cleido mas	29.1/45.4	29.3/45.8	29.8/46.8	30.8/48.8	32.5/52.4	34.9/58.2	38.4/62.3		
Deltoid	32.9/44.4	32.9/44.5	32.9/44.5	32.9/44.6	33.0/44.8	33.0/45.1	33.1/45.6	33.2/46.1	33.3/46.9
Biceps	29.5/45.2	29.6/45.2	29.6/45.4	29.8/45.7	30.1/46.2	30.5/46.9	31.0/48.0		
Ext dig comm	34.9/50.0	34.9/50.1	35.1/50.5	35.4/51.3	35.9/52.5	36.6/54.4	37.7/57.2	39.1/61.1	40.9/66.5
Abd digit V	44.4/63.5	44.7/64.0	45.2/65.5	46.4/68.6	48.2/73.9	51.0/82.7	54.8/96.6		
Quadriceps	35.9/47.9	36.0/48.0	36.5/48.2	37.5/48.5	39.0/49.1	41.3/50.0	44.6/51.2		
Ant tibialis	49.4/80.0	49.3/79.8	49.2/79.3	48.9/78.3	48.5/76.8	47.9/74.5	47.0/71.4	45.8/67.5	44.3/62.9
